# Availability, Accessibility, and Suitability of Native Flowers from Central Chile to *Mastrus ridens*, a Parasitoid of Codling Moth

**DOI:** 10.3390/insects16070665

**Published:** 2025-06-26

**Authors:** Tania Zaviezo, Alejandra E. Muñoz, Erick Bueno

**Affiliations:** Facultad de Agronomía y Sistemas Naturales, Pontificia Universidad Católica de Chile, Avda. Vicuña Mackenna 4860, Macul, Santiago 7820436, Chile; aemunoz@uc.cl (A.E.M.); ebbueno@uc.cl (E.B.)

**Keywords:** agroecology, biodiversity, conservation biological control, flower, natural enemy, nectar

## Abstract

**Simple Summary:**

Non-crop vegetation can be used to conserve pest natural enemies by providing complementary food, and by using native plants, biodiversity conservation in agricultural areas can be promoted. The potential for nectar provision of 13 flowering species native to Chile, and two introduced, was studied considering *Mastrus ridens*, a natural enemy of the codling moth. Nectar availability was studied by looking at flowering periods, accessibility by contrasting the size of the flowers and head of the insect, and suitability by the longevity when exposed to nectar solutions or cut flowers. Most species had long flowering periods, potentially making nectar available when insects are active, but they differed in nectar accessibility and profitability. Of the 13 native species, nectar was easily accessible for *M. ridens* in only four species. Parasitoid longevity did not increase with nectar solutions, but they lived longer with cut flowers of two native species, *Teucrium bicolor* and *Sphaeralcea obtusiloba,* and one introduced, *Fagopyrum esculentum*, making them candidates for *M. ridens* conservation. In conclusion, using suitable native flowering species in agroecosystems can improve codling moth control and biodiversity conservation.

**Abstract:**

Habitat manipulation through non-crop vegetation management is a strategy in conservation biological control, and using native plants is attractive because they can also help in biodiversity conservation. The potential for nectar provision of 13 flowering species native to Chile, and two introduced, was evaluated considering *Mastrus ridens* (Hymenoptera: Braconidae). Nectar availability was studied through flower phenology, accessibility through flower and parasitoid morphology, and suitability through longevity when exposed to nectar solutions or cut flowers. Most species had long flowering periods, potentially making nectar available when adults are active, but they differed in nectar accessibility and profitability. Of the 13 native species, nectar was easily accessible for *M. ridens* in *Cistanthe grandiflora, Sphaeralcea obtusiloba, Andeimalva chilensis,* and *Lycium chilense*. None of the nine native species tested with nectar solutions increased longevity, but with cut flowers, parasitoids lived longer with the natives *Teucrium bicolor* and *S. obtusiloba*, and the introduced *Fagopyrum esculentum*, making them candidates for *M. ridens* conservation. Females lived longer with cut flowers of *T. bicolor* and *S. obtusiloba* than with their nectar solutions. In conclusion, using the native flowering species *Teucrium bicolor* and *Sphaeralcea obtusiloba* in agroecosystems can serve biological control and biodiversity conservation.

## 1. Introduction

Conservation biological control aims to preserve natural enemies in agroecosystems to increase their impact on pest control [1]. Various strategies can be implemented, among which, habitat manipulation through non-crop vegetation management is probably the most widely used [2,3,4,5]. Still, its formal study is relatively recent and lacking in many parts of the world, particularly when using native plants [6,7]. Non-crop plants used for conservation biological control can provide many different alternative or complementary resources for natural enemies, including structural (e.g., refuge, overwintering sites) and trophic (e.g., pollen, nectar, alternative prey) [5,8]. The benefits of providing these resources on longevity or fecundity have been demonstrated for many species in the laboratory and the field (e.g., [9,10,11,12,13,14,15,16,17]). Most generalist predators can profit from a range of resources provided by the non-crop vegetation, such as alternative prey, protein in the form of pollen, sugars from nectar or honeydew, and refuge from disturbances or for overwintering (e.g., [9,13,17,18,19,20]). Nevertheless, in the case of hymenopteran parasitoids, the range of alternative resources needed is reduced, being mostly restricted to sugars, given that most adult parasitoids depend primarily or solely on carbohydrates for energy [21,22]. Many studies have proved that when adult parasitoids receive sugar meals, longevity and fecundity can be greatly enhanced in laboratory conditions (e.g., [10,23,24,25,26,27,28]). In the field, sugar is provided mainly by nectar (floral and extrafloral) and/or by honeydew secreted by hemipteran species [12,13,29,30,31,32,33,34]. Notwithstanding, the amount and types of sugars provided by these sources vary greatly, and they do not have the same nutritional value for different species of parasitoids, with some having no effects or even being detrimental (e.g., [10,24,27,28,32,35,36,37,38]).

Many field studies have looked at the effects of adding nectar sources nearby or within crops on parasitoid abundance and pest control, but very few have investigated if nectar feeding actually occurs (e.g., [14,30,39,40]). In several systems, a positive effect of adding nectar sources in parasitism has been found (e.g., [10,11,41,42]), yet in many others, no increase in parasitism has been noted (e.g., [43,44]). This may be due to many different factors, including dispersal away from the nectar sources after sugar feeding [45], low survival because of pesticide use, nectar-positive effects on other trophic levels (i.e., herbivorous pest, hyperparasitoids) [46,47,48,49], other sources of sugars already available (e.g., weeds, honeydew [50]) or lack of feeding by the parasitoids on the nectar sources provided (reviewed by [51]). In this last aspect, it is important to note that for parasitoids to be able to feed from the nectar of a flower of a given species, first it must be available (i.e., flowering and nectar production must occur when parasitoids are active), it should be accessible (i.e., parasitoids should be able to reach the nectar) [52,53,54], and flowers should be attractive [2,24,52,55]. Additionally, for the parasitoid to benefit from nectar feeding, it must be suitable (i.e., have high nutritional value) (e.g., [53]). All the factors mentioned above highlight the importance of carefully studying plant traits and the interaction with the parasitoid species that is aimed to be conserved, before recommending flowering species for a conservation biological control program (e.g., [40,52,53,56,57]).

Using native plants to provide nectar sources for natural enemies is an attractive idea because they are well-adapted to local conditions and can provide additional ecosystem services, such as biodiversity conservation. Blaauw and Isaacs [58] studied native North American perennial plants to enhance the control of blueberry pests, finding higher egg predation in fields adjacent to the flower plantings. Pandey et al. [59] studied the longevity of parasitoids of brassica pests exposed to flowers of native Australian plants in the laboratory, finding positive effects with several of them, although some also benefited herbivores. In Chile, even with few studies on the use of non-crop plants for conservation biological control [60], none are with native plants aiming at nectar provision for natural enemies. This is surprising since central Chile is the main area of intensive agricultural production in the country and is also where Chile’s main hotspot of vascular flora is located, known as the “Hotspot of Central Chile” [61]. This is also the country’s main area for fruit production, including apples and walnuts https://catastro-fruticola-inicio-esri-ciren.hub.arcgis.com/ (accessed on 17 May 2025).

*Mastrus ridens* Horstmann (Hymenoptera: Ichneumonidae) is an important parasitoid of codling moth (*Cydia pomonella* L.), a lepidopteran pest native to central Asia and present in most parts of the world where apples and walnuts are grown, including Chile [62,63,64]. The parasitoid is from the same region of origin as the pest (southern Kazakhstan and north-western China), and its biology corresponds to a specialist gregarious ectoparasitoid that attacks mature codling moth larvae in their cocoons [65,66,67,68]. This species is also synovogenic, i.e., it matures eggs during its adult life, and does not resorb them, suggesting the need for sugar meals for energy to forage in this life stage [26]. *Mastrus ridens* has been used in classical biological control programs against codling moth in several countries [69]. Nevertheless, it has had variable results after its release in the different regions, which might be related to the genetic diversity of populations released and the presence of complementary sex determination (CSD), because low genetic diversity results in a lower proportion of females in the progeny [69,70,71]. This, in turn, hinders population increase and parasitism and could result in lower pest control. Other factors could also play a role in the lower performance of this parasitoid in some areas, like the availability of sugar resources, particularly in commercial orchards. Previous studies have demonstrated that in the laboratory, providing diluted honey increases *M. ridens* survival by 5 to 8-fold (e.g., [23,26]), and thus in orchards with low vegetational diversity and limited availability of suitable flowers or low-quality nectar, this species is probably sugar-limited, affecting its establishment and impact.

The objective of this study was to determine which species of native plants from central Chile can provide sugar resources that benefit *M. ridens* and have the potential to be incorporated in conservation biological control programs for codling moth, while also contributing to biological conservation. We hypothesized that native floral species provide nectar as a complementary resource for *M. ridens*, but that there would be differences in usefulness among plant species according to their nectar availability, accessibility, and suitability for the parasitoid. Accordingly, we evaluated nectar availability through flower phenology, nectar accessibility through flower and parasitoid morphology, and suitability through its impact on parasitoid longevity.

## 2. Materials and Methods

### 2.1. Parasitoid Populations Rearing

*Mastrus ridens* used for the experiments came from a colony maintained in our laboratory (Facultad de Agronomía y Sistemas Naturales, Pontificia Universidad Católica de Chile, Santiago, Chile), descending from a mix of those collected in Kazakhstan (Almaty and nearby locations) in 2013 (four locations) and 2015 (two locations), and insects from a New Zealand mass-rearing colony imported to our laboratory in 2014 (details in [69]). Parasitoid populations were reared in a room at approximately 25 ± 2 °C, 40 ± 10% RH, and 16 h light: 8 h dark photoperiod, in fine mesh cages (BugDorm-4F3030, MegaView Science Co., Ltd., Taichung, Taiwan), provided with water and honey solution. In these cages, non-diapausing 5th instar cocooned codling moth (*C. pomonella*) larvae from the laboratory culture (reared on Stonefly Heliothis Diet; Ward’s Natural Science, Rochester, NY, USA), were introduced in 2- to 3-cm wide strips of corrugated cardboard. To obtain insects for the experiments, cardboards exposed to the parasitoids for 2 weeks in the cages were inspected for larvae parasitism, and *M. ridens* cocoons were collected and placed individually in vials until adult emergence.

### 2.2. Plant Species Selection

The flowering plant species studied were selected to be perennial, native to Chile (with several endemic to central Chile), and well adapted to the climatic conditions of central Chile, where apple is grown. In total, 13 species belonging to seven different families with varying characteristics were selected (Table 1, Figure S1). For the nectar accessibility and parasitoid longevity experiments, two non-native species, widely studied in conservation biological control projects [3], were also included: buckwheat (*Fagopyrum esculentum* Moench, Polygonaceae) and Alyssum (*Lobularia maritima* (L.) Desv., Brassicaceae).

### 2.3. Nectar Availability

Nectar availability was estimated through the flowering period of each species, which was determined by following plant phenology approximately every other week. Three potted plants per species (except for *Plectocephalus chilensis*, where only two were available), grown at the University Campus (−33.49642, −70.60898, Santiago, Chile), were followed from September 2023 to January 2025. Additionally, established plants (two per species) grown in Pumahuida nursery (−33.35390, −70.69100, Huechuraba, Santiago, Chile) were followed from October 2023 to March 2024, which is the growing season in the southern hemisphere and when parasitoids are active. At each observation date, plants’ phenological stages were classified as vegetative (no flower buds, actively growing), pre-flowering (flower buds present, but not open), or flowering (open flowers present). Then, combining years and locations, the proportion of observations with flowering plants of the total observations of a given month was estimated for each species. *Andeimalva chilensis* phenology was not included because it was not available in the first year of the experiments.

### 2.4. Nectar Accessibility for M. ridens

Nectar accessibility was estimated by measuring the head width (between the extreme lateral margins of the eyes, Figure S2) of *M. ridens* females (n = 44) and males (n = 16) and morphological characteristics of the flowers to determine the ‘effective flower depth’, defined as “the distance between the opening that is still accessible by the insect’s head and the top of the nectaries” [54]. For flowers, first, the position of the nectaries was determined under a dissecting scope (Leica EZ4 D). Then, the maximum and minimum width of the corolla and the distance from the corolla opening to the observed location of the nectaries at the corolla base (corolla length) were measured (Figure S3). For larger flowers, a digital caliper was used (Figure S3a), and for smaller flowers, a picture was taken under a dissecting scope and analyzed using ImageJ software version 1.54g [78], with a scale of 1:0.0027 (pixel/millimeter). For Asteraceae, these measures were taken at the beginning of the corolla tube of the individual tubular flowers (florets), which form the dense inflorescences (Figure S3b). Effective flower depth was the distance between *M. ridens’* head in its deepest possible position within the flower and the nectar source. Effective flower depth was 0 when *M. ridens* could freely reach the nectaries (mean head width narrower than the minimum width of the corolla), and it was equal to corolla length when the mean head width was wider than the maximum width of the corolla opening. In the intermediate case, i.e., when the mean head width was narrower than the maximum width of the corolla but wider than the minimum corolla width, it was estimated using the formula in van Rijn et al. [54].

### 2.5. Nectar Suitability

Nectar suitability was measured through its effects on parasitoid female longevity. We conducted two experiments, each using a different technique, one exposing females to nectar solutions and the other using cut flowers.

#### 2.5.1. Nectar Solutions

The solutions were prepared by gently shaking 5 fresh flowers or inflorescences, consecutively, for 1 min in a vial containing 1 mL of distilled water (adapted from [79]). Flowers were cut from the plants between 9:30 and 11:30 am, so they were completely open with a low chance of being fed upon, and were immediately brought to the laboratory to prepare the solutions. For the experiments, newly emerged (<6 h) *M. ridens* females were put in 30 mL vials with a few drops of the nectar solution. The drops were set with a brush on the wall of the vial two times per day, between 10:00 and 11:00 a.m. and between 15:00 and 16:00 p.m. In total, 9 native flower species were tested: *Erigeron luxurians* (n = 6), *Haplopappus chrysanthemifolius* (n = 4), *Senecio eruciformis* (n = 7), *Encelia canescens* (n = 19), *Teucrium bicolor* (n = 21), *Andeimalva chilensis* (n = 5), *Sphaeralcea obtusiloba* (n = 9), *Cistanthe grandiflora* (n = 9), and *Lycium chilense* (n = 14). Also, the non-natives buckwheat (*F. esculentum*) and Alyssum (*L. maritima*) (10 replicates each) were used as reference because they have been widely used in studies with other parasitoids. Negative (distilled water in a piece of cotton at the bottom of the vial, n = 28) and positive (50% diluted honey placed on the wall of vials with a brush, n = 30) controls were also included. Experiments were run from March 2023 to January 2025, according to insect and flower availability, with positive and negative controls interspaced. Vials with the females and the different treatments were kept in a growing chamber at approximately 25 ± 2 °C, 40 ± 10% RH, with 16 h light and 8h dark photoperiod, and female survival was checked daily.

#### 2.5.2. Cut Flowers

Plants and flower buds were marked and followed to select flowers with 2–3 days of anthesis for the experiments, increasing the chance of having a good amount of nectar production. The day before flowers were used for the experiments, they were bagged with a fine mesh to exclude flower-visiting insects and allow nectar accumulation. The following day, between 10:00 and 11:00, open flowers or inflorescences were cut from the plant. Then, in the laboratory, the flower’s stem was put in a small Eppendorf tube (1.5 mL) filled with distilled water, with the lid cut but sealed around the stem with Parafilm paper. The tube with the flower was put inside a plastic container (5 cm high by 2 cm in diameter) attached to the interior walls with tape. A mesh was used at the top of the container to avoid insect escape while allowing ventilation. For the experiments, newly emerged (<6 h) *M. ridens* females were put in the containers with the flowers. Flowers were replaced with freshly cut ones every 2 or 3 days to expose parasitoids to fresh flowers throughout their lifetime. In total, 8 native flower species were tested: *E. luxurians* (n = 8), *H. chrysanthemifolius* (n = 10), *S. eruciformis* (n = 3), *E. canescens* (n = 19), *T. bicolor* (n = 13), *A. chilensis* (n = 10), *S. obtusiloba* (n = 13), and *Lycium chilense* (n = 20). *Cistanthe grandiflora* was not included because its flowers wilted very quickly once cut from the plant. As with the nectar solution experiment, buckwheat (n = 19), Alyssum (n = 14), negative controls (distilled water, n = 40), and positive controls (50% diluted honey, n = 37) were included. Experiments were run from January 2023 to January 2025, according to insect and flower availability, with positive and negative controls interspaced. Containers with the parasitoids and flowers were kept in a growing chamber at approximately 25 °C, 40% RH, and 16:8 L/D photoperiod, and female survival was checked daily.

#### 2.5.3. Statistical Analysis

Parasitoid female longevity with nectar solutions and with cut flowers, along with their corresponding control treatments, were analyzed using generalized linear models (GLM), with a Poisson distribution and log link function. Additionally, to study whether the technique of providing nectar to females affected their longevity, for the 10-flower species in which nectar solutions and cut flowers were used, a GLM with Poisson distribution and log link function was used, with flower species and technique as fixed effects. Models were evaluated for overdispersion using the deviance ratio. All models were analyzed using ANOVA tables based on F-test statistics. Mean differences were analyzed using Fisher LSD (*p* < 0.05) test with the Benjamini and Hochberg correction for controlling false discovery rate [80]. Statistical analyses were performed using InfoStat version 2020 [81] and R version 4.0.4. Means presented in figures and text are adjusted means of untransformed data ± standard error.

## 3. Results

### 3.1. Nectar Availability

**Figure 1:**
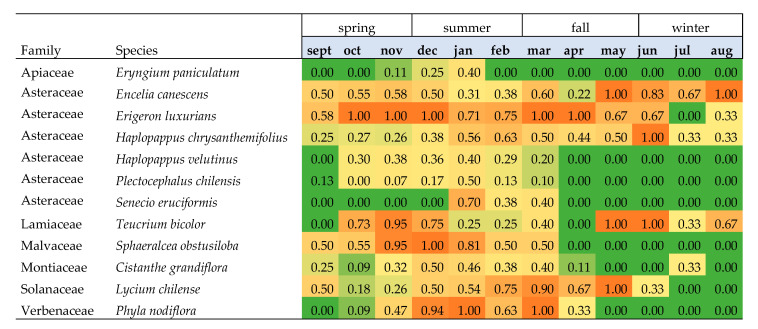
Plant species flowering phenology. Numbers indicate the proportion of observations with flowering plants of the total observations of a given month (combining years and locations). Orange to green gradients indicate in orange the largest proportion of observations with flowering plants, in yellow when 50% of the observations had flowering plants, and in green when the lowest proportion of observations had flowering plants and therefore were in the vegetative stage.

Most species had long flowering periods, beginning in the middle of the spring and through the summer, coinciding with *M. ridens* adult activity. The species with the longest flowering period were *E. luxurians* and *E. canescens*, having 10 and 9 months with at least 50% of plants flowering, respectively. The species with the shortest flowering period were *S. eruciformis*, *E. paniculatum*, and *P. chilensis*, with 3, 2, and 1 months with at least 25% of plants flowering, respectively (Figure 1).

### 3.2. Nectar Accessibility for M. ridens

**Figure 2:**
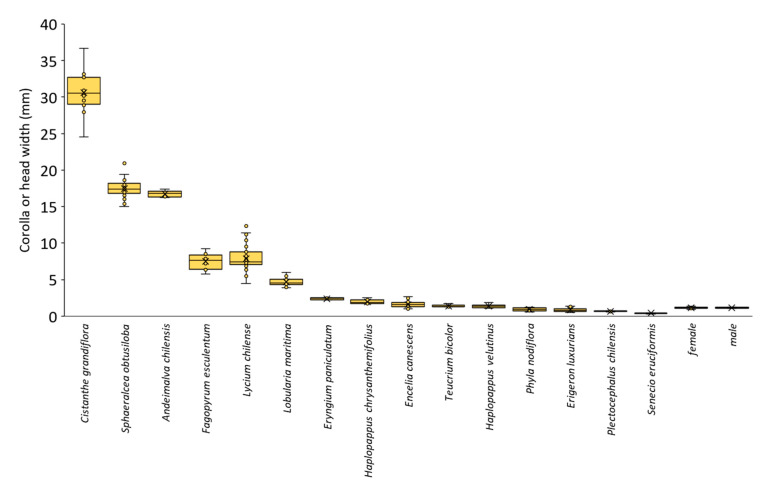
Box plot of corolla maximum width of 13 native plant species from Chile, two introduced, and head width of female and male *Mastrus ridens*.

**Figure 3:**
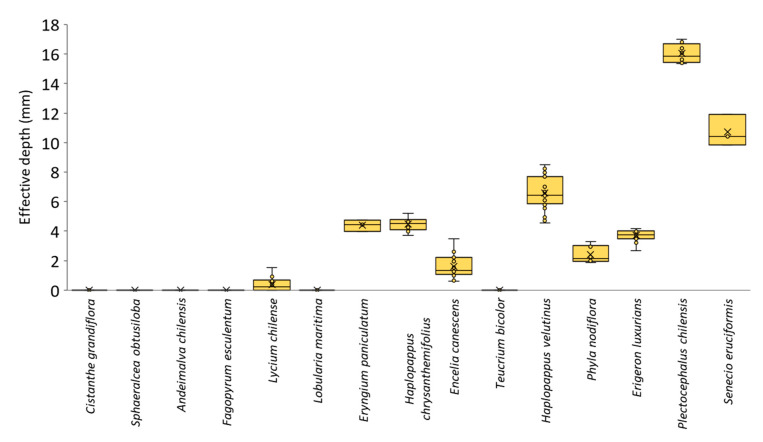
Box plot of the estimated effective flower depth for *Mastrus ridens* of 13 plant species native to Chile and two introduced.

Parasitoid head width was (mean ± SEM) 1.157 ± 0.017 mm (range: 0.9–1.4) for females and 1.175 ± 0.028 mm (range: 1.0–1.4) for males. Corolla maximum width varied greatly among plant species, from 30.65 ± 0.87 mm in *C. grandiflora* to 0.39 ± 0.02 mm in *S. eruciformis*. In 11 out of the 15 plant species, *M. ridens* head fit through the corolla opening (95% confidence intervals did not overlap) (Figure 2, Table S1). For the four species in which *M. ridens’* head could not fit (*P. nodiflora*, *E. luxurians*, *P. chilensis*, and *S. eruciformis*), effective depth was considered equal to the corolla length, ranging from 2.39 ± 0.24 to 16.04 ± 0.64 mm (Figure 3, Table S2).

Corolla minimum width varied from 8.40 ± 0.30 mm in *C. grandiflora* to 0.13 ± 0.01 mm in *S. eruciformis* (Table S2). In six plant species, *M. ridens’* head fit all the way through the corolla and reached the nectaries, including the natives *C. grandiflora*, *S. obtusiloba*, *A. chilensis*, and *T. bicolor*, and the non-natives *F. esculentum* and *L. maritima*. For these species, the effective depth was considered to be 0 mm (Figure 3).

In the remaining five species (*E. paniculatum*, *H. chrysanthemifolius*, *E. canescens*, *H. velutinus*, *L. chilense*), *M. ridens’* head fit at the opening of the corolla but could not freely reach the nectaries, at least in some flowers. The estimated effective depth for these species was between 0.37 ± 0.07 and 6.60 ± 0.23 mm (Figure 3, Table S2).

### 3.3. Nectar Suitability: Parasitoid Longevity

#### 3.3.1. Nectar Solutions

**Figure 4:**
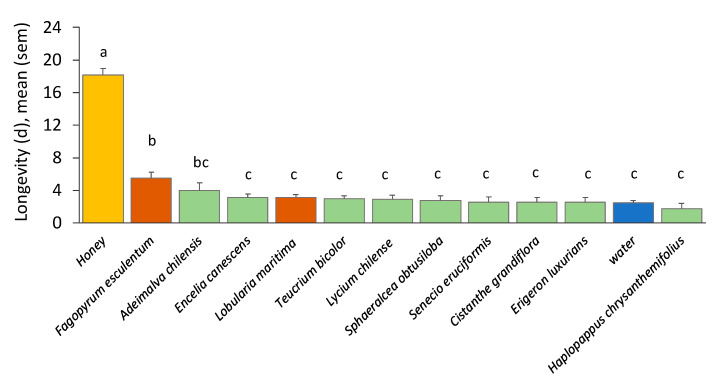
*Mastrus ridens* female longevity (days, mean ± s.e.) when exposed to nectar solutions from flowers of Chilean native species (green bars), introduced plant species (red bars), and positive (50% honey solution, yellow bar) and negative (water, blue bar) controls). Different letters above the bars denote significant differences in means (Fisher LSD, *p* < 0.05, test with Benjamini and Hochberg correction).

There was a significant effect of treatment in *M. ridens* female longevity when exposed to nectar solutions, honey solution, or water (F (12, 159) = 65.09, *p* < 0.0001). Females lived the longest with honey solution (18.2 ± 0.8 d), followed by *F. esculentum* (buckwheat, 5.5 ± 0.7 d). With the rest of the nectar solutions, *M. ridens* lived between 4.0 ± 0.9 d with *A. chilensis* and 1.8 ± 0.7 d with *H. chrysanthemifolius*, but none of them differed significantly from the longevity with water (2.4 ± 0.3 d; Figure 4).

#### 3.3.2. Cut Flowers

**Figure 5:**
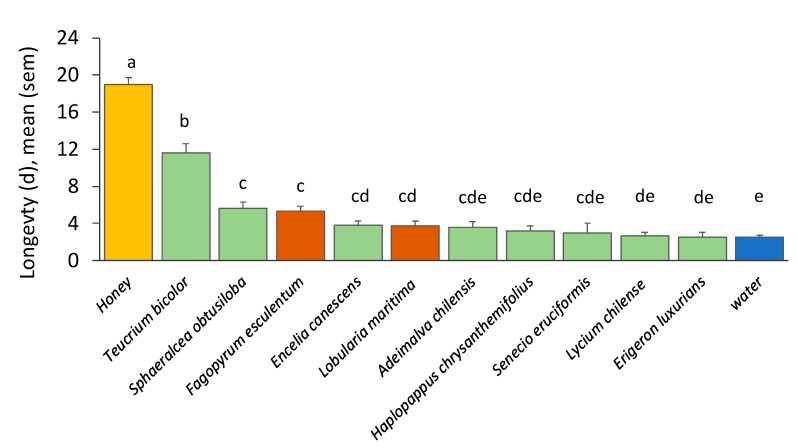
*Mastrus ridens* female longevity (days, mean ± s.e.) when exposed to cut flowers from plants native to Chile (green bars), introduced plant species (red bars), and positive (50% honey solution, yellow bar) and negative (water, blue bar) controls. Different letters above the bars denote significant differences in means (Fisher LSD, *p* < 0.05, test with Benjamini and Hochberg correction).

There was a significant effect of treatment in *M. ridens* female longevity when exposed to cut flowers, honey solution, or water (F (11, 194) = 80.45, *p* < 0.0001). Females lived the longest with honey solution (19.0 ± 0.7 d) and the least with water (2.5 ± 0.3 d). With five flower species, females lived significantly longer than with water: the natives *T. bicolor* (11.6 ± 1.0 d), *S. obtusiloba* (5.6 ± 0.7 d), and *E. canescens* (3.8 ± 0.5 d) and the non-natives *F. esculentum* (5.3 ± 0.5 d) and *A. maritimus* (3.7 ± 0.5 d) (Figure 5). With the flowers of the other species, females lived between 3.6 ± 0.6 d and 2.5 ± 0.6 d, not differing from the longevity with water (Figure 5).

#### 3.3.3. Nectar-Providing Technique

There was a significant effect of the nectar technique (F (1, 211) = 11.13, *p* = 0.001), plant species (F (9, 211) = 7.33, *p* < 0.0001), and technique × species (F (9, 211) = 6.07, *p* < 0.0001) on female longevity. When nectar was provided in cut flowers, on average, females lived one day longer than with nectar solutions (4.0 ± 0.2 and 3.0 ± 0.2 d, respectively). Considering both methods combined, females lived the longest with *T. bicolor* and *F. esculentum* (5.7 ± 0.4 and 5.4 ± 0.5 d, respectively), followed by *S. obtusiloba* (4.0 ± 0.5 d). The remaining seven species lived between 3.8 ± 0.5 d and 2.4 ± 0.5 d, being significantly less than *T. bicolor* and *F. esculentum*. When considering plant species and technique, females exposed to flowers of *T. bicolor* lived more than three times longer than with nectar solutions of this species (10.8 ± 0.1 and 3.0 ± 0.4 d, respectively), and *S. obtusiloba* twice as long with flowers than with nectar solutions (5.6 ± 0.7 and 2.8 ± 0.6 d, respectively). With the rest of the plant species, female longevity was similar with both techniques (Table S3).

## 4. Discussion

In this study, we evaluated whether the flowers of native plant species from central Chile can provide sugar resources that benefit *M. ridens*, a specialist parasitoid of the codling moth, to select those with the most potential for conservation biological control programs targeting this key pest of apples and walnuts, while also contributing to biological conservation within an agricultural area. For this, we compared the nectar availability, accessibility, and suitability for the parasitoid of 13 native plant species, and also included two non-native species widely used in these studies. As expected, not all flowering species were equally beneficial for *M. ridens*. After considering all three requirements, only one native species showed high potential for its use in conservation biological control of this parasitoid species, and it was much better than the two popular non-native species.

Regarding nectar availability, 9 of the 12 native species followed had long flowering periods, spanning most of the spring and summer, potentially being a source of sugars for the parasitoid. A more precise measure of nectar availability would require determining flower abundance, by area or plant, and the amount each produces [82]. In the plant phenology assessments, the number of flowers by plant was counted, which can help improve the estimate of nectar availability. Nevertheless, one of the most complex and demanding tasks remaining is the estimation of nectar volume produced by individual flowers for each species. In insect-pollinated plants, this volume typically falls below 1 μL per day [83], making accurate measurement particularly challenging for the minute flowers of families such as Apiaceae and Asteraceae. In addition, nectar production and secretion are also determined by flower duration and age (e.g., [84,85,86]), and environmental variables, including temperature, soil water content, wind speed, and relative humidity (e.g., [87]). Information on some of these flower traits might be available for the flora of well-studied regions or particular species (e.g., [82,88]), and potentially in public databases, such as those contributing to the TRY Plant Trait Database [89], but this is not the case for most of the native and endemic flora of Chile. Nevertheless, floral counts, along with flower size, can be a good enough proxy for nectar availability in many cases [82,90,91].

Nectar accessibility depends on flowers and insect morphology [54,92]. For most parasitoid species, due to their short mouthparts, head width, along with the position of the nectaries in the flower, corolla width, form, and depth are the key traits determining nectar accessibility, and thus, the range of flowers that can potentially provide nectar to a given parasitoid species. The plant species tested showed important differences in nectar accessibility for *M. ridens* and, of the 13 native species evaluated, nectar was easily accessible in only four: *C. grandiflora*, *S. obtusiloba*, *A. chilensis*, and *T. bicolor*. In two other native species, nectar could probably be accessible, at least in larger flowers or for smaller insects, as their mean effective flower depth was 0.37 and 1.62 mm (*L. chilense* and *E. canescens*, respectively). A study of nectar accessibility for the syrphid fly *Episyrphus balteatus* found that in flowers with depths beyond 2 mm, survival was always low, but the critical depth was about half for Asteraceae species [54]. In our study, except for *E. canescens*, the effective flower depth for the Asteraceae species tested was larger than 3.5 mm, while all the species from other families had lower values. This finding was somewhat surprising because Asteraceae is the family most used in conservation biological control studies [3,7]. For the two non-native species, *F. esculentum* and *L. maritima*, the effective flower depth for *M. ridens* was zero, meaning their nectar was easily accessible. Given their flower morphology and nectaries’ position, the nectar of these species is accessible to many natural enemies, and this might partly explain why they have been used in many conservation biological control projects around the world (e.g., [3] and references therein, [16,57]). Another important aspect concerning nectar accessibility is that some plants also produce extrafloral nectar (e.g., on leaves, stems, stipules, or bracts), representing a highly accessible source of sugars, given the presence of exposed nectar droplets [93]. No glands or extrafloral nectar production was observed in the plant species studied, but more detailed observations should be carried out to rule out their presence.

Although a flower may offer accessible nectar to natural enemies, its consumption is not necessarily beneficial and may even be detrimental to certain species (e.g., [16,24,27,38,94]). This variable outcome is related to nectar composition, such as amount and type of sugars, other nutrients (e.g., amino acids), and detrimental components, like defensive chemicals (e.g., alkaloids, glycosides, saponins) (reviewed by [13]). In this study, none of the nine native species tested in the experiments with nectar solutions resulted in female longevity significantly better than when provided with water. Only with the nectar solution of the introduced *F. esculentum* plant did females have significantly increased longevity, by about 2.3 times, but this was still only one-third of their longevity with the honey solution. On the other hand, when females were exposed to cut flowers, with three native plants and two introduced, they lived significantly more than with water, yet significantly less than with honey solution. Among these plant species, *F. esculentum* and *S. obtusiloba* doubled female lifetime (from 2.5 days with water to about 5 days), and only with *T. bicolor* was it above 10 days, making them good candidates for a conservation biological control program for *M. ridens*. These results agree with several others where only a subset of the plant species tested were beneficial (e.g., [16,53,57]), and contrast with the few where most flower species increased longevity of parasitoids when compared to water (e.g., [95]). Also, the results indicate that, among the introduced species, *F. esculentum* is more suitable than *L. maritima* for *M. ridens*, as is also the case in studies with some other parasitoid species (e.g., [53,95,96,97]).

When comparing the nectar-providing techniques, females lived much longer with cut flowers of *T. bicolor* and *S. obtusiloba* than with their respective nectar solutions, while for the other eight species, the longevity was similar with both techniques. This was an unexpected result because for the flowers where *M. ridens* could not fit its head, the nectar solutions would make it accessible, and for the flowers where the head fit, accessing the nectar source would require some energy expense, and thus, we expected longevity with nectar solutions to be greater for most species. The observed result, particularly for *T. bicolor* and *S. obtusiloba*, suggests that the nectar extraction technique used was not very effective, or that it might have diluted the sugars too much. Extracting nectar from flowers that produce very small volumes is an important challenge, and several techniques have been suggested, the most common being the use of microcapillary tubes, filter paper, and washing or rinsing flowers with distilled water [79,83]. In this study, the washing technique was selected because other techniques were not feasible for the smaller flowers, and a standard method for all species was needed. Additionally, in a study comparing these techniques using flowers of *Eucalyptus spathulate* (Myrtaceae), more sugars were extracted when using the washing or rinsing techniques, with the former being the most practical in the field [79]. Nevertheless, it would be interesting to study in more detail how different techniques can influence the amount of sugars and other components collected for the species of this study, and to relate them to the longevity of natural enemies. On the other hand, this study supports the utility of using cut flowers (with their peduncles in water) as a reliable methodology to test flower suitability in laboratory conditions, which is a common technique in these studies, with practical advantages over using intact flowers still attached to the plant [95]. In a previous study testing the flower presentation method, which included *F. esculentum* and *L. maritima* among others, found that cut flowers and intact flowers had the same effect on the longevity of the parasitoid *Aphidius ervi* (Hymenoptera: Braconidae), showing that there is not a large detrimental effect on nectar flow rates, concentration, or composition when removing them from the plants [95]. Nevertheless, in some species, using cut flowers could be problematic. For example, in this study, we could not include *C. grandiflora* in the cut flower experiment because they wilted very quickly in the experimental conditions.

One additional aspect important to understanding the interactions between flower resources and natural enemies, besides availability, accessibility, and suitability, is flower attractiveness and the cues (e.g., volatile and visual) and the potential role of nectar-inhabiting microorganisms in modulating these responses [52,54,55]. Knowing and selecting for these traits in flowers will ensure that the natural enemies can locate the chosen species and frequently visit them in the field. Something similar has been proposed to assign a predictive “pollination value” of flora for pollinators, where floral traits associated with a flower’s visual attractiveness (size, color, and UV reflection) are mentioned, in addition to traits associated with accessibility (botanical family, symmetry, and shape), and reward (pollen and nectar quantity and quality) [98]. Consequently, to complete our evaluation of the native flower species, we are currently conducting attractiveness studies with *M. ridens* and the most promising species according to the experiments presented here, including how the parasitoid response associates with volatile and visual characteristics of the flowers. Finally, the potential positive effect of the flower resources on the pest should also be assessed [40,46,47,49,97,99,100], and studies on the effect of the native flowers selected on the longevity and fecundity of codling moth are also underway.

## 5. Conclusions

In this study, we showed that after considering the availability, accessibility, and suitability of 13 native flowers from central Chile to *M. ridens*, only one species, *T. bicolor* (Lamiaceae), showed very good potential for use in a conservation biological control program, with another species, *S. obtusiloba* (Malvaceae), showing a modest potential. Also, from the two non-native species widely used in these projects, only *F. esculentum* showed modest potential, while *L. maritima* was not suited. These results support the idea that when a particular natural enemy is the target for conservation, flower traits must be carefully studied, and evaluations must be carried out before the establishment of plant species in the field. We also provide evidence that some native flowers have the potential to benefit non-native natural enemies and even outperform standard introduced plant species. Consequently, using suitable native flowering species in agricultural areas can serve both biological control and native biodiversity conservation and potentially provide other ecosystem services.

## Figures and Tables

**Table 1:** List of native species (Family and Scientific name) studied with their life forms, floral arrangement, flower type, and biogeographic origin according to [72,73,74,75,76,77]. Images of the flowers are in the Supplementary Materials, Figure S1.

Family/Scientific Name	Life Form	Flower Arrangement	Flower Type	Biogeographic Origin
Apiaceae				
* Eryngium paniculatum* Cav. & Dombey ex F. Delaroche	Herb	Dense inflorescence	Tubular	Native (Chile, Argentina)
Asteraceae				
* Encelia canescens* Lam.	Subshrub	Dense inflorescence	Ligulate and tubular	Native (Bolivia, Chile, Galápagos, Peru)
* Erigeron luxurians* (Skottsb.) Solbrig	Subshrub	Dense inflorescence	Ligulate and tubular	Endemic (Central Chile)
* Haplopappus velutinus* J. Remy	Shrub	Dense inflorescence	Ligulate and tubular	Native (Chile, Argentina)
* Haplopappus chrysanthemifolius* (Less.) DC	Shrub	Dense inflorescence	Ligulate and tubular	Endemic (Central Chile)
* Plectocephalus chilensis* (Bertero ex Hook. & Arn.) G. Don	Subshrub	Dense inflorescence	Ligulate and tubular	Endemic (Northern and Central Chile)
* Senecio eruciformis* J. Remy	Subshrub	Dense inflorescence	Ligulate and tubular	Native (Chile, Argentina)
Lamiaceae				
* Teucrium bicolor* Sm.	Subshrub	Loose inflorescence	Bilabiate	Endemic (Central Chile)
Malvaceae				
* Sphaeralcea obtusiloba* (Hook.) G. Don	Subshrub	Loose inflorescence	Campanulate	Endemic (Central Chile)
* Andeimalva chilensis* (Gay) J.A. Tate	Shrub	Loose inflorescence	Campanulate	Endemic (Northern and Central Chile)
Montiaceae				
* Cistanthe grandiflora* (Lindl.) Schltdl.	Herb	Solitary	Campanulate	Endemic (Central Chile)
Solanaceae				
* Lycium chilense* var. *confertifolium* (Miers) F.A. Barkley	Shrub	Solitary	Campanulate	Native (Argentina, Chile)
Verbenaceae				
* Phyla nodiflora* var. *minor* (Gillies & Hook.) N.O’Leary & Múlgura	Subshrub	Dense inflorescence	Ligulate and tubular	Native (Argentina, Bolivia, Brazil, Chile, Ecuador, Paraguay, Peru, Uruguay)

## Data Availability

The raw data supporting the conclusions of this article will be made available by the authors on request.

## Supplementary Materials

The following supporting information can be downloaded at https://www.mdpi.com/article/10.3390/insects16070665/s1, Figure S1: Family and Scientific name of the native species used in the study and pictures of representative flowers. Figure S2: Example of how head width was measured; Figure S3. Examples of flower measurements. Table S1: Descriptive statistics of corolla maximum width of 13 native plant species from Chile and two non-natives, and the head width of *M. ridens* females and males (mm). Table S2: Minimum corolla width, corolla length, and estimated effective depth (mm) for *M. ridens* for the 14 species studied. Table S3: *Mastrus ridens* female longevity (days, mean ± s.e.) when exposed to cut flowers and nectar solutions of ten flower species.
