# Supplementary material for: Availability, Accessibility, and Suitability of Native Flowers from Central Chile to Mastrus ridens, a Parasitoid of Codling Moth
Source: Insects. 2025 Jun 26;16(7):665. doi: 10.3390/insects16070665 (PMC12295075; doi:10.3390/insects16070665)

Table S1. Descriptive Statistics of corolla maximum width of 13 native plant species from Chile and two non-natives (from widest to narrowest), and the head width of *M. ridens* females and males (mm). Plants are arranged from the widest to the narrowest.

Species				95% Confidence Interval Mean				
	N	Mean	Std. Error of Mean	Upper	Lower	Std. Deviation	Minimum	Maximum
C. grandiflora	12	30.652	0.874	32.576	28.729	3.028	24.580	36.700
S. obtusiloba	35	17.499	0.236	17.979	17.018	1.398	15.000	21.300
A. chilensis	5	16.730	0.200	17.285	16.175	0.447	16.250	17.400
L. chilense	31	7.881	0.311	8.516	7.245	1.733	4.500	12.400
F. esculentum	9	7.468	0.371	8.324	6.612	1.114	5.800	9.200
L. maritima	15	4.699	0.168	5.060	4.339	0.651	3.900	6.000
E. paniculatum	3	2.400	0.100	2.830	1.970	0.173	2.200	2.500
H. chrysanthemifolius	10	1.960	0.083	2.148	1.772	0.263	1.700	2.500
E. canescens	26	1.600	0.080	1.764	1.436	0.406	1.000	2.600
T. bicolor	5	1.400	0.077	1.615	1.185	0.173	1.300	1.700
H. velutinus	25	1.364	0.044	1.455	1.273	0.220	1.100	1.900
P. nodiflora	6	0.925	0.089	1.154	0.696	0.218	0.600	1.200
E. luxurians	31	0.839	0.041	0.922	0.756	0.226	0.500	1.300
P. chilensis	8	0.676	0.020	0.722	0.630	0.055	0.580	0.750
S. eruciformis	8	0.388	0.018	0.431	0.344	0.052	0.280	0.450
M. ridens female	44	1.157	0.017	1.192	1.122	0.115	0.900	1.400
M. ridens male	16	1.175	0.028	1.235	1.115	0.113	1.000	1.400

Table S2. Minimum corolla width, corolla length, and estimated effective depth (mm) for *M. ridens* for the 14 species studied. Species are arranged according to estimated effective depth (from smallest to largest).

Species	Minimum corolla width			Corolla length			Estimated effective depth		
	N	mean	s.e.	N	mean	s.e.	N	mean	s.e.
C. grandiflora	2	8.40	0.30	12	19.04	1.06	12	0.00	0.00
S. obtusiloba	8	4.30	0.23	30	16.35	0.23	35	0.00	0.00
A. chilensis	5	3.57	0.32	5	15.62	0.32	5	0.00	0.00
F. esculentum	5	1.75	0.06	5	1.30	0.06	9	0.00	0.00
L. maritima	5	1.46	0.05	10	1.51	0.10	15	0.00	0.00
T. bicolor	5	1.41	0.09	5	3.92	0.12	5	0.00	0.00
L. chilense	31	1.04	0.06	31	6.45	0.25	31	0.37	0.07
E. canescens	26	0.43	0.01	26	2.95	0.11	26	1.62	0.14
P. nodiflora	3	0.49	0.04	6	2.72	0.29	6	2.39	0.24
E. luxurians	31	0.42	0.01	31	3.69	0.06	31	3.69	0.06
E. paniculatum	3	0.30	0.13	3	4.39	0.22	3	4.39	0.22
H. chrysanthemifolius	10	0.62	0.03	10	8.56	0.25	10	4.46	0.14
H. velutinus	25	0.62	0.01	25	8.36	0.06	25	6.60	0.23
S. eruciformis	5	0.13	0.01	8	10.28	0.47	8	10.28	0.47
P. chilensis	5	0.35	0.03	8	16.04	0.23	8	16.04	0.64

Table S3. *Mastrus ridens* female longevity (days, mean ± s.e.) when exposed to cut flowers and nectar solutions of ten flower species. Means followed by the same letters do not statistically differ (Fisher LSD; $p < 0.05$, with Benjamini and Hochberg correction for controlling false discovery rate.

Nectar-providing technique	Plant species	mean	s.e.	Means comparisons
Cut flower	*T. bicolor*	10.83	0.95	a
Cut flower	*S. obtusiloba*	5.62	0.66	b
Nectar solution	*F. esculentum*	5.50	0.74	bc
Cut flower	*F. esculentum*	5.32	0.53	bc
Nectar solution	*A. chilensis*	4.00	0.89	bcd
Cut flower	*E. canescens*	3.84	0.45	bcd
Cut flower	*L. maritima*	3.71	0.52	bcd
Cut flower	*A. chilensis*	3.67	0.64	bcd
Cut flower	*H. chrysanthemifolius*	3.20	0.57	bcd
Nectar solution	*E. canescens*	3.11	0.40	bcd
Nectar solution	*L. maritima*	3.00	0.55	bcd
Cut flower	*S. eruciformis*	3.00	1.00	bcd
Nectar solution	*T. bicolor*	2.95	0.37	cd
Nectar solution	L. chilense	2.93	0.46	cd
Nectar solution	*S. obtusiloba*	2.78	0.56	cd
Cut flower	L. chilense	2.70	0.37	cd
Nectar solution	E. *luxurians*	2.60	0.72	cd
Nectar solution	*S. eruciformis*	2.57	0.61	d
Cut flower	E. *luxurians*	2.50	0.56	d
Nectar solution	*H. chrysanthemifolius*	1.75	0.66	d

Figure S1. Family and Scientific name of the native species used in the study and pictures of representative flowers.

Family/Scientific name		
**Apiaceae**		Picture credits
Eryngium paniculatum Cav. & Dombey ex F. Delaroche	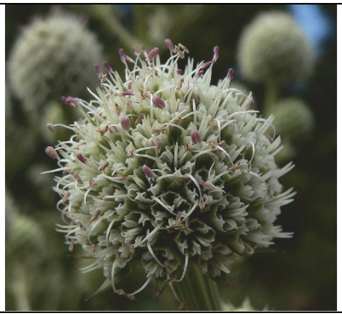	Stan Shebs, San Francisco Botanical Garden
**Asteraceae**		
Encelia canescens Lam.	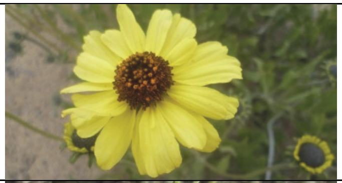	Monica Musalem, Vivero Pumahuida
Erigeron luxurians (Skottsb.) Solbrig	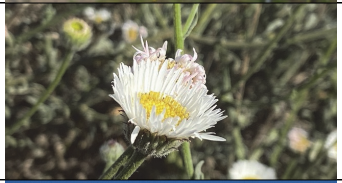	Monica Musalem, Vivero Pumahuida
Haplopappus velutinus J. Remy	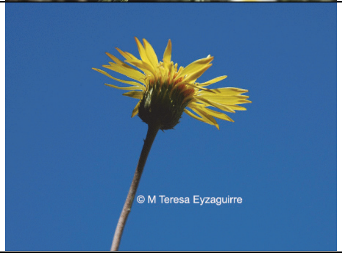	M Teresa Eyzaguirre, Fundación RA Philippi

Haplopappus chrysanthemifolius (Less.) DC	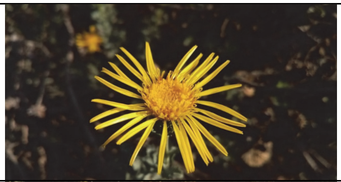	Monica Musalem, Vivero Pumahuida
Plectocephalus chilensis (Bertero ex Hook. & Arn.) G. Don	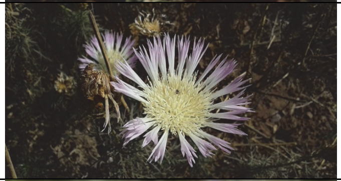	Monica Musalem, Vivero Pumahuida
Senecio eruciformis J. Remy	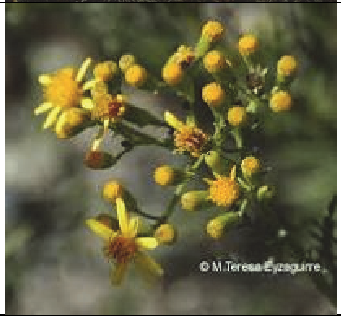	M Teresa Eyzaguirre, Fundación RA Philippi

**Lamiaceae**		
Teucrium bicolor Sm.	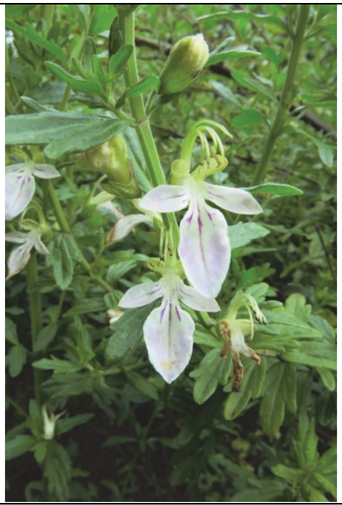	M.F. Gardner, Royal Botanical Garden Edinburg,
**Malvaceae**		
Sphaeralcea obtusiloba (Hook.) G. Don	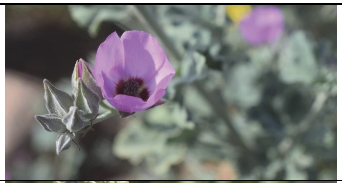	Monica Musalem, Vivero Pumahuida
Andeimalva chilensis (Gay) J.A. Tate	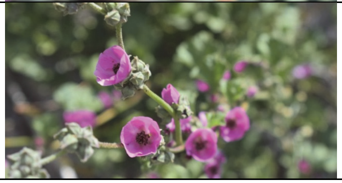	Monica Musalem, Vivero Pumahuida

**Montiaceae**		
Cistanthe grandiflora (Lindl.) Schltdl.	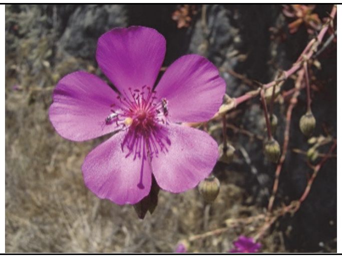	Monica Musalem, Vivero Pumahuida
**Solanaceae**		
Lycium chilense var. *confertifolium* (Miers) F.A. Barkley	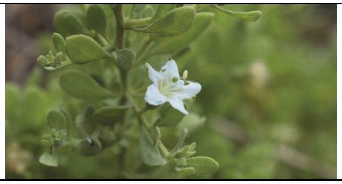	Monica Musalem, Vivero Pumahuida
**Verbenaceae**		
Phyla nodiflora var. *minor* (Gillies & Hook.) N.O'Leary & Múlgura	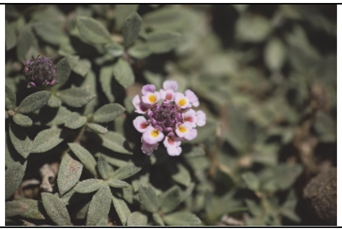	Steven Bodzin, iNaturalisy

Figure S2. Example of how head width was measured (white line): distance between the extreme lateral margins of the eyes.

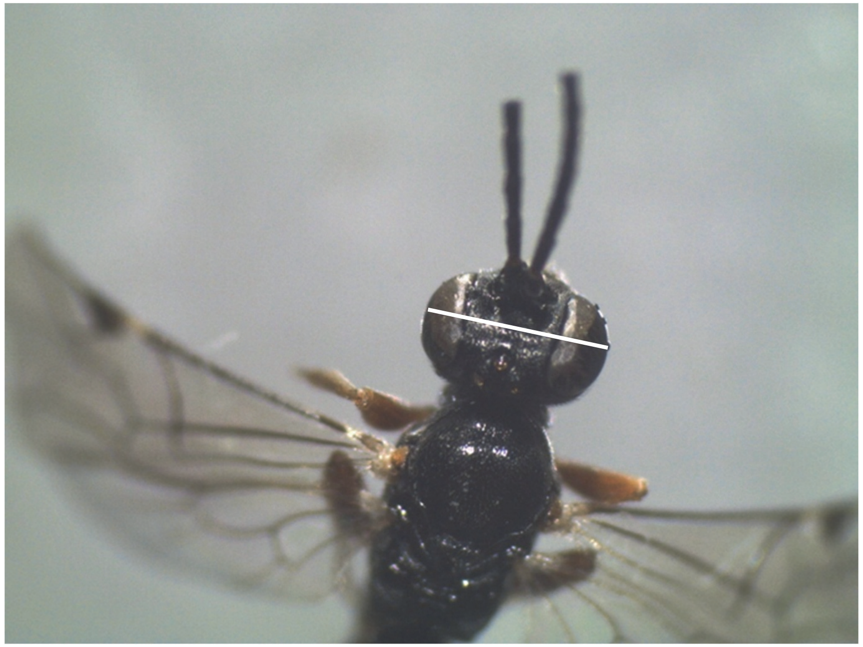

Figure S3. Examples of flower measurements: 1 = maximum width of the corolla; 2 = corolla length (distance from the corolla opening to the observed location of the nectaries); 3 = minimum width of the corolla

a) *Sphaeralcea obtusiloba*, where parasitoid head width was narrower than the minimum width of the corolla. Effective depth was 0. For larger flowers a digital caliper was used for some measures.

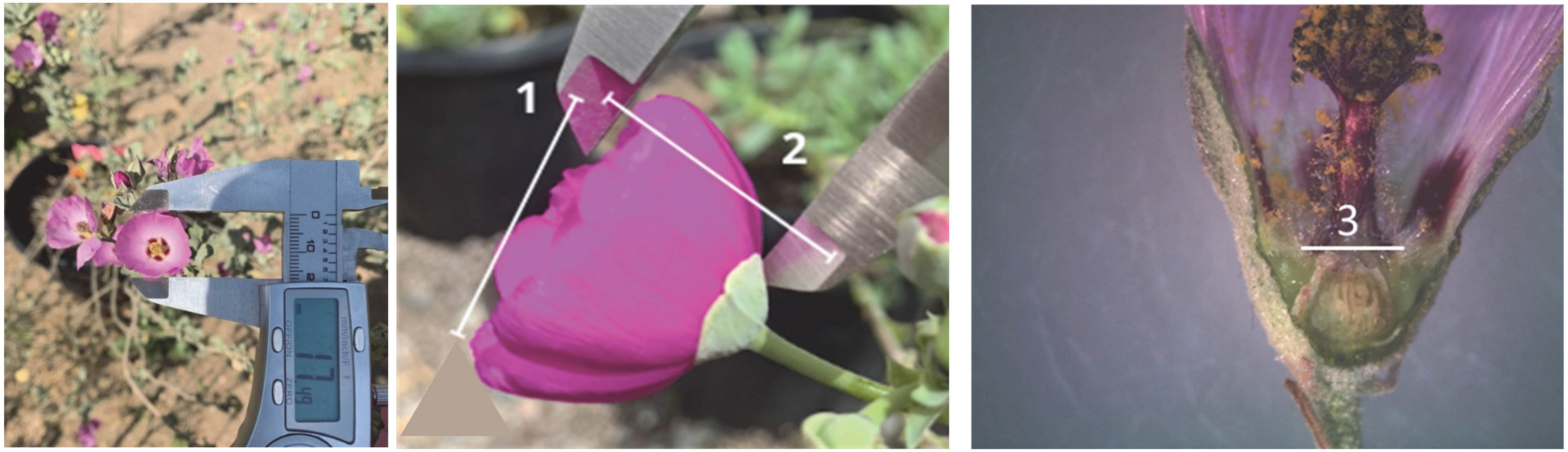

b) *Erigeron luxurians*, the parasitoid head width was wider than the maximum width of the corolla opening. Effective flower depth was equal to corolla length.

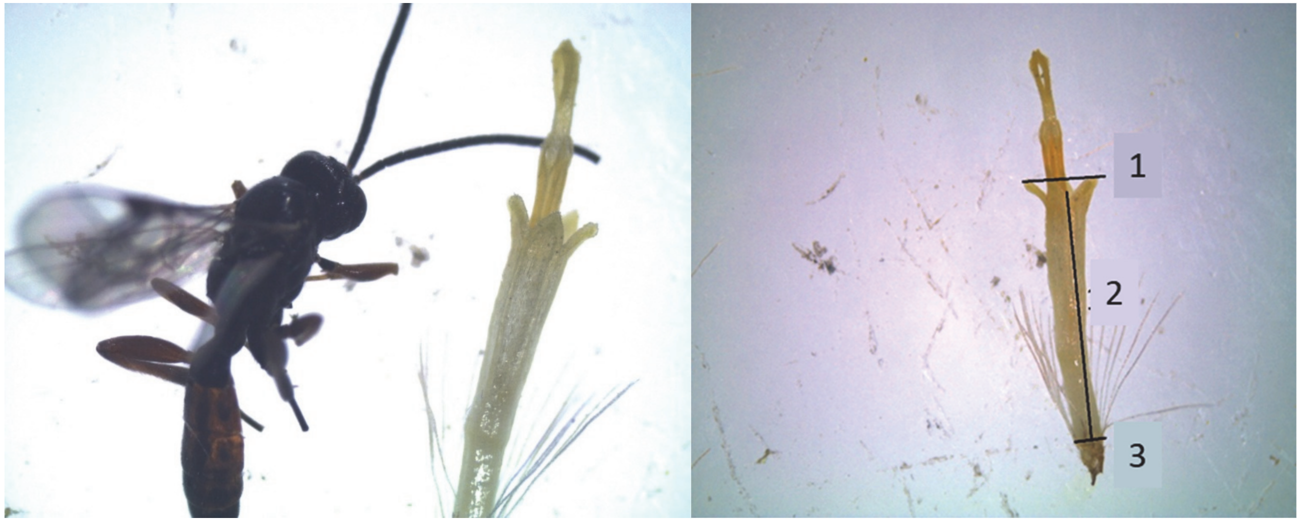

c) *Lycium chilense,* where mean head width was narrower than the maximum width of the corolla but wider than the minimum corolla width, and effective flower depth was estimated using van Rijn et al. [56] formula.

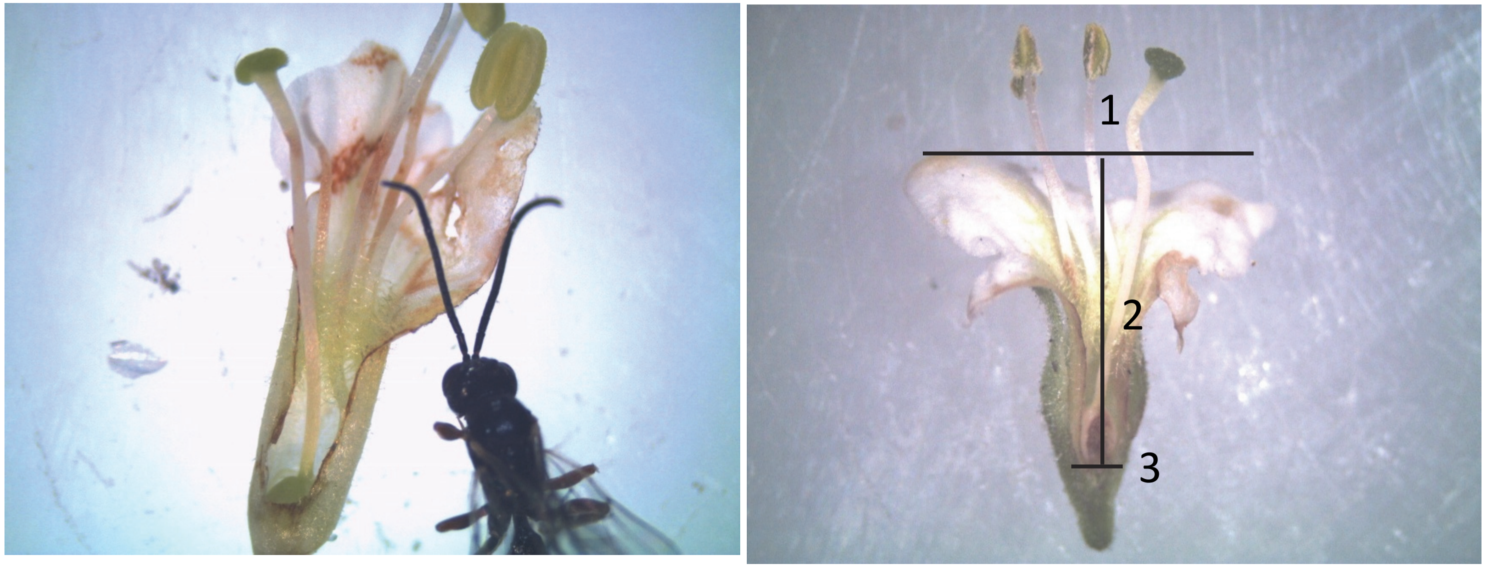

Supplement: Supplementary file 1 [file insects-16-00665-s001.zip › insects-3685632-supplementary.pdf]
